# Supplementary material for: Wealth disparities and economic flow: Assessment using an asset exchange model with the surplus stock of the wealthy
Source: PLoS One. 2021 Nov 4;16(11):e0259323. doi: 10.1371/journal.pone.0259323 (PMC8568155; doi:10.1371/journal.pone.0259323)
Supplement: Attachment — Submitted filename: Response to Reviewers.doc [file pone.0259323.s001.doc]

October xx, 2021

Rodrigo Huerta-Quintanilla

Academic Editor

*PLOS ONE*

Dear Editor and Reviewers:

Thank you for your suggestions and comments on our manuscript titled “Wealth disparities and economic flow: assessment using an asset exchange model with the surplus stock of the wealthy.” We also appreciate the time and effort you and each of the reviewers have dedicated to providing insightful feedback on ways to strengthen our paper. We have incorporated changes that reflect the detailed suggestions you have so graciously provided. We hope that our edits and the responses provided below satisfactorily address all the issues and concerns you and the reviewers have noted.

To facilitate your review of our revisions, the following is a point-by-point response to the questions and comments delivered in your letter [PONE-D-21-26966].

**Response to Editor:**

1. One point that want to mention is that equations are not introduced or commented properly along the paper. The reader expects also that variables and parameter are defined in the text when the reading of the material is clearer.

**Response:** Thank you for helpful suggestion. We have added equations (1) to (3) and an explanation about variables and parameters (p. 7, line 150, p.8, line 161, and p.9, line 173).

**Response to Reviewer #1:**

1. The introduction section should be extended. It is too abstract. The authors should introduce the aim and objective of the research and make a presentation why is there a need for their research.

**Response:** Thank you for this suggestion. We have added the aim and objectives (p. 3, lines 57–61) as well as information about the content of the paper (p. 4, lines 79–87).

2. The main idea of the article should be carefully explained, as it is hardly legible in its present form.

**Response:** Thank you for this suggestion. As written in our response to the editor above, we have added equations (1) to (3) and an explanation about the variables and parameters (pp. 7–9).

3. The author should extend consider introducing a literature review section and survey more literature related to these aspects; an important benchmark for research is simply missing. Therefore, the literature list should be supplemented with selected items in this field and discussed in the literature review.

**Response:** Thank you for this suggestion. We have added a literature review section (pp. 5–7).

5. The figures are poorly visible due to low resolution.

**Response:** Thank you for this suggestion. All figures are now in high resolution (600 dpi).

6. Consider combining the Results and Discussion section and discuss the results on an ongoing basis.

**Response:** Thank you for this suggestion. We have re-edited the discussion section (pp. 22–25) so that the discussion more naturally follows the results.

7. It is necessary to designate a section of conclusions clearly indicating the obtained results of the analysis.

**Response:** Thank you for this suggestion. We have added a conclusions section (pp. 25–27) and re-edited the content of the previous draft’s discussion and conclusions.

**Response to Reviewer #2:**

1. Wonder whether in the introduction the Authors could add some more references on the already knowledge on latest high-level papers.

**Response:** Thank you for this suggestion. As noted in the response to reviewer #1 above, we have added a literature review section (pp. 5–7).

2. Include an elaborate discussion explaining the exact significance of your findings.

**Response:** Thank you for this suggestion. As written in response to reviewer #1 above, we have separated the discussion and conclusion sections, and re-edited them to explain the significance of our findings in the conclusion (pp. 25–27).

3. Probably the quality of the pictures in figure 1 could be improved in the last version.

**Response:** Thank you for this suggestion. As noted in response to reviewer #1 above, all figures are now in high resolution (600 dpi).

Thank you, once again, for giving us the opportunity to strengthen our manuscript with your valuable comments and queries. We have worked hard to incorporate your feedback and hope that these revisions persuade you to accept our submission.

Sincerely,

Takeshi Kato

Hitachi Kyoto University Laboratory, Open Innovation Institute, Kyoto University

Yoshidahonmachi, Sakyo-ku, Kyoto-shi, Kyoto 606-8501, Japan

+81-75-753-9716

kato.takeshi.3u@kyoto-u.ac.jp
